# Improvement of cardiac function by placenta-derived mesenchymal stem cells does not require permanent engraftment and is independent of the insulin signaling pathway

**DOI:** 10.1186/scrt490

**Published:** 2014-08-21

**Authors:** Juliana A Passipieri, Tais H Kasai-Brunswick, Grazielle Suhett, Andreza B Martins, Guilherme V Brasil, Dilza B Campos, Nazareth N Rocha, Isalira P Ramos, Debora B Mello, Deivid C Rodrigues, Beatriz B Christie, Bernardo J Silva-Mendes, Alex Balduíno, Renato M Sá, Laudelino M Lopes, Regina C Goldenberg, Antonio C Campos de Carvalho, Adriana B Carvalho

**Affiliations:** Instituto de Biofísica Carlos Chagas Filho, Universidade Federal do Rio de Janeiro, Av Carlos Chagas Filho 373, Sala G2-053, Rio de Janeiro, RJ 21941-902 Brazil; Instituto Nacional de Cardiologia, Rua das Laranjeiras 374, Rio de Janeiro, 22240-006 Brazil; Departamento de Radiologia, Hospital Universitário Clementino Fraga Filho, Rua Rodolpho Paulo Rocco 255, Rio de Janeiro, 21941-913 Brazil; Universidade Federal Fluminense, Rua Professor Hernani Melo 101, Niterói, 24210-130 Brazil; Instituto Nacional de Ciência e Tecnologia de Biologia Estrutural e Bioimagem, Av Carlos Chagas Filho 373, Rio de Janeiro, 21941-902 Brazil; Centro de Pesquisa, Tecnologia e Inovação, Universidade Veiga de Almeida, Rua Ibituruna 108, Rio de Janeiro, 20271-020 Brazil; Centro Pré-Natal de Diagnóstico e Tratamento, Clínica Perinatal, Rua das Laranjeiras 445, Rio de Janeiro, 22240-002 Brazil; Department of Obstetrics and Gynecology, Western University, London Health Sciences Centre-Victoria Hospital, B2-401, London, ON N6H 5W9 Canada

## Abstract

**Introduction:**

The objective of this work was to evaluate the efficacy of placenta-derived mesenchymal stem cell (MSC) therapy in a mouse model of myocardial infarction (MI). Since MSCs can be obtained from two different regions of the human term placenta (chorionic plate or villi), cells obtained from both these regions were compared so that the best candidate for cell therapy could be selected.

**Methods:**

For the *in vitro* studies, chorionic plate MSCs (cp-MSCs) and chorionic villi MSCs (cv-MSCs) were extensively characterized for their genetic stability, clonogenic and differentiation potential, gene expression, and immunophenotype. For the *in vivo* studies, C57Bl/6 mice were submitted to MI and, after 21 days, received weekly intramyocardial injections of cp-MSCs for 3 weeks. Cells were also stably transduced with a viral construct expressing luciferase, under the control of the murine stem cell virus (MSCV) promoter, and were used in a bioluminescence assay. The expression of genes associated with the insulin signaling pathway was analyzed in the cardiac tissue from cp-MSCs and placebo groups.

**Results:**

Morphology, differentiation, immunophenotype, and proliferation were quite similar between these cells. However, cp-MSCs had a greater clonogenic potential and higher expression of genes related to cell cycle progression and genome stability. Therefore, we considered that the chorionic plate was preferable to the chorionic villi for the isolation of MSCs. Sixty days after MI, cell-treated mice had a significant increase in ejection fraction and a reduction in end-systolic volume. This improvement was not caused by a reduction in infarct size. In addition, tracking of cp-MSCs transduced with luciferase revealed that cells remained in the heart for 4 days after the first injection but that the survival period was reduced after the second and third injections. Quantitative reverse transcription-polymerase chain reaction revealed similar expression of genes involved in the insulin signaling pathway when comparing cell-treated and placebo groups.

**Conclusions:**

Improvement of cardiac function by cp-MSCs did not require permanent engraftment and was not mediated by the insulin signaling pathway.

**Electronic supplementary material:**

The online version of this article (doi:10.1186/scrt490) contains supplementary material, which is available to authorized users.

## Introduction

Multipotent mesenchymal stem cells (MSCs) are an attractive source of stem cells for tissue repair. They are known for their immunomodulatory properties [1] and ability to differentiate into several mesenchymal lineages, including adipocytes, osteocytes, and chondrocytes [2], when submitted to specific culture conditions. They have been identified in various organs [3–5], but frequency and differentiation potential of adult MSCs are dependent upon the age of the donor [6, 7]. Moreover, invasive procedures may be required to obtain them.

On the other hand, fetal MSCs can be derived from the fetus or from extra-embryonic structures that are of fetal origin [8]. These structures are discarded after birth and therefore are easy to obtain and available in large scale, which makes them interesting sources for the isolation and banking of stem cell populations. In this context, MSCs phenotypically similar to bone marrow MSCs have been isolated from various extra-embryonic structures, including amniotic fluid [9, 10], Wharton’s jelly [11], amniotic and chorionic membrane [12], and human placenta, which has been used by many authors for the isolation of stem cells (Additional file 1).

Several reports have shown that it is possible to extract MSCs from both the chorionic villi (cv) [13–22] and chorionic plate (cp) [22–27] of the term placenta. However, it remains undetermined whether there is any difference between cv-MSCs and cp-MSCs or which placental region is a better source of extra-embryonic MSCs. In the present study, we described and compared the isolation and phenotypic and functional characterization of cell populations derived from these regions of the human term placenta to investigate which would be a preferable source of MSCs for cell therapy.

In addition, cell therapy using MSCs has emerged as a promising alternative to treat chronic diseases. This is especially important in the case of ischemic heart disease and congestive heart failure, which are major causes of morbidity and mortality throughout the world and impose a significant economic burden on most health systems [28]. Initially, regeneration of cardiac muscle was thought to be the main mechanism involved in the improvement of cardiac function promoted by cell therapy [29–32]. However, this capacity has been challenged, especially in the case of adult stem cells [33–37]. In this work, we evaluated the role of placenta-derived MSCs in the treatment of cardiac dysfunction post-myocardial infarction (MI) in immunocompetent mice.

## Materials and methods

### Isolation and culture of human placenta-derived mesenchymal stem cells

Full-term human placentas (38 to 40 weeks of gestation; n = 16) were obtained after maternal informed consent. All experiments were approved by our local institutional review board (Hospital Universitário Clementino Fraga Filho, Universidade Federal do Rio de Janeiro, Rio de Janeiro, Brazil). Once the amniotic membrane was manually separated and discarded, the harvested pieces of chorionic plate and chorionic villi were mechanically minced and enzymatically digested with 100 and 200 U/mL of type II collagenase, respectively (Roche, Basel, Switzerland), for 5 hours at 37°C with orbital shaking. Both single-cell suspensions were filtered through a 100-μm cell strainer (BD Biosciences, San Jose, CA, USA) to eliminate undigested fragments, and the cells were collected by centrifugation at 670*g* for 15 minutes. Mononuclear cells were recovered by Ficoll density gradient (Ficoll-Paque PLUS-1077 g/mL; Amersham Bioscience, now part of GE Healthcare, Little Chalfont, Buckinghamshire, UK) after centrifugation at 400*g* for 30 minutes at room temperature. Cells were suspended in expansion medium constituted by alpha modified essential medium (αMEM) (LGC Biotecnologia, São Paulo Brazil) supplemented with 15% fetal bovine serum (FBS) (Gibco, now part of Invitrogen Corporation, Carlsbad, CA, USA) and 1% penicillin/streptomycin (Gibco), plated at a density of 5 × 10^5^ cells per centimeter squared, and incubated at 37°C in a 5% CO_2_ incubator. Cells were allowed to adhere for 2 days, and the non-adherent cells were washed away during medium changes. Third-passage cells were used in all experiments, unless otherwise specified.

### Fibroblast colony-forming unit assay

Fibroblast colony-forming units (CFU-F) assays were performed on freshly isolated cells from chorionic plate (n = 6) and chorionic villi (n = 5). Each well of a six-well plate (Corning Costar, Tewksbury MA, USA) received 10^5^ freshly isolated cells, which were cultured in expansion medium supplemented with 10^-5^ M of hydrocortisone (Strides Arcolab Limited, Bangalore, Karnataka). After a 15-day period, colonies were fixed with methanol PA (Vetec Quimica Fina, now part of Sigma-Aldrich, St. Louis, MO, USA) for 5 minutes at room temperature and counted after Giemsa staining (Proquimios, Rio de Janeiro, Brazil).

### Flow cytometry

For immunophenotypic analysis, third-passage cp-MSCs (n = 5) and cv-MSCs (n = 4) were dissociated and suspended in blocking solution containing cold phosphate-buffered saline (PBS) supplemented with 5% FBS for 20 minutes. All antibodies, except CD133 (Miltenyi Biotec, Bergisch Gladbach, Germany), were purchased from BD Biosciences.

The following antibodies conjugated with fluorescein isothiocyanate (FITC), phycoerythrin (PE), peridinin chlorophyll protein (PerCP), allophycocyanin (APC), or phycoerythrin-Cy7 (PE-Cy7) were used: CD45 (clone 2D1), CD34 (clone 8Y12), CD44 (clone L178), CD117 (clone 104D2), CD31 (clone WM59), CD133 (clone 29C3), CD166 (clone 3A6), CD54 (clone HA58), CD146 (clone P1H12), CD14 (clone MΦP9), CD19 (clone 4G7), HLA-DR (clone TU36), CD73 (clone AD2) and CD90 (clone 5E10), and CD105 (clone 266). Cells were stained in accordance with the instructions of the manufacturer, and 7 aminoactinomyocin D (7AAD) was added to exclude dead cells.

After 30 minutes of incubation at 4°C, cells were washed with PBS, centrifuged at 400*g* for 3 minutes, and suspended in PBS for acquisition. Samples were acquired in a BD FACSCanto flow cytometer (BD Biosciences), and the resulting data were analyzed by using FACSDiva software version 6.1.1 (BD Biosciences).

### Multipotent differentiation assays

All differentiation reagents were purchased from Sigma-Aldrich, unless otherwise specified. Media were changed three times a week, and no passages were made during the differentiation protocols. Non-induced control samples were submitted to the same procedures but were maintained in expansion medium.

Adipogenic differentiation was induced by culturing cp-MSCs (n = 3) and cv-MSCs (n = 3) for 21 days in Dulbecco’s modified Eagle’s medium-high glucose (DMEM-HG) supplemented with 10% FBS, 1% penicillin/streptomycin, 10^-6^ M dexamethasone, 10 μg/mL human insulin, 0.5 μM isobutylemethylxanthine, and 200 μM indomethacin. Cells were fixed with formalin for 30 minutes at room temperature, and cytoplasmic lipid droplets were stained with 0.2% Oil Red O.

Osteogenic differentiation was performed by culturing cp-MSCs (n = 3) and cv-MSCs (n = 3) in DMEM-HG, 10% FBS, 1% penicillin/streptomycin, 10^-6^ M dexamethasone, 10 mM β-glycerolphosphate, and 0.5 μM ascorbic phosphate acid for 21 days. Cells were fixed with formalin for 30 minutes at room temperature, and extracellular calcium deposits were stained after 30 minutes of incubation with 1% Alizarin Red in water.

Chondrogenic differentiation was performed under micromass conditions by using a STEMPRO^®^ Chondrogenesis Differentiation kit (catalog A10071-01; Invitrogen Corporation) in accordance with the guidelines of the manufacturer. Briefly, micromasses were generated by using approximately 8 × 10^4^ cp-MSCs (n = 3) and cv-MSCs (n = 3) seeded in 5-μL droplets of cell solution and cultured for 2 hours under high-humidity conditions. Micromasses were subsequently cultured in chondrogenesis medium (Invitrogen Corporation) for 21 days. The pellets were fixed with 4% paraformaldehyde for 30 minutes at room temperature and embedded in paraffin. The presence of proteoglycans, characteristic of chondrocytes, was evaluated after staining with 1% Alcian Blue in 3% acetic acid solution for 30 minutes. Counterstaining was performed with Nuclear Fast Red.

### Population doubling time

Growth kinetics of cp-MSCs (n = 3) and cv-MSCs (n = 5) was assessed by estimating population doubling time (PDT). Cells (10^4^) were plated in 35-mm cell culture dishes with a 2-mm grid (Nalge Nunc International, Penfield, NY, USA) in expansion medium. Cells were counted daily, and the number of cells per millimeter squared was calculated. These values were used to build a cells per millimeter squared versus time plot. Appling a base 2 logarithm in the cells per millimeter squared axis, we were able to perform a linear regression, in which the inverse of the angular coefficient α was used to calculate PDT.

### Karyotype analysis

For detection of aneuploidy, chromosome preparations were performed in cp-MSCs (n = 4) and cv-MSCs (n = 3). Cells were arrested in metaphase with 1.2 μg/mL of colcemide (Sigma-Aldrich), dissociated from culture flasks with trypsin-EDTA (Gibco), and incubated with hypotonic solution (KCl 75 mM) (Vetec Quimica Fina) for 15 minutes at 37°C. Cells were collected after centrifugation (200*g* for 10 minutes) and fixed with a methanol-acetic acid solution (3:1). Chromosome spreads were obtained by pipetting suspension drops onto clean glass slides. Metaphase cells were GTG-banded by using Giemsa, and 20 metaphases were karyotyped for each sample.

### Animals

Experiments were performed in 8- to 10-week-old female immunocompetent C57Bl/6 mice (20.5 to 25.0 grams). All experiments were performed in conformity with the *Guide for the Care and Use of Laboratory Animals* (National Institutes of Health) and were approved by the Committee on the Ethics of Animal Use of the Federal University of Rio de Janeiro under number IBCCF 026. Mice were housed at a controlled temperature (23°C) with a 12:12-hour light-dark cycle and received standard mouse chow and water *ad libitum*.

### Myocardial infarction, cp-MSCs injection, and echocardiography

MI was induced as previously described [38]. Briefly, animals (n = 19) were anesthetized with an intraperitoneal injection of xylazine (20 mg/kg) and ketamine (80 mg/kg). After endotracheal intubation and ventilation (Harvard Apparatus, Holliston, MA, USA), left anterior thoracotomy was performed, and the left anterior descending coronary artery (LAD) was permanently ligated. A sham-operated group (n = 11) was prepared in a similar manner but without tightening the suture around the LAD.

Cardiac function parameters—ejection fraction (EF), end-systolic volume (ESV), and end-diastolic volume (EDV)—were determined by using Simpson’s method at baseline and at 20 days post-MI by echocardiographic imaging (Vevo 770, probe 30.0 MHz; VisualSonics, Toronto, ON, Canada).

At 21 days after MI, 30 μL of a 25% solution of Matrigel (BD Biosciences) in PBS containing either 10^5^ cp-MSCs (n = 11) or no cells (n = 8) was injected directly into the border zone of the infarcts by using an echocardiography-guided procedure. Mice received a weekly injection for 3 weeks. Cardiac function parameters were analyzed by echocardiogram on days 7, 15, 23, and 39 after the first injection (Additional file 2). All analyses were performed by a blinded investigator.

### Construction of lentiviral vector containing Luc2 gene and production of lentiviral particles

Lentiviral vector pMSCV.luc2.T2A.Puro was constructed by cloning the luciferase 2 (Luc2) gene into the commercial vector pCDH.MCS.T2A.Puro-MSCV (System Biosciences, Mountain View, CA, USA). Luc2 gene was polymerase chain reaction (PCR)-amplified from pGL4.50 plasmid (Promega Corporation, Madison, WI, USA) by using primers forward 5′-*GCTAGC*GAATTC**GCCACC**ATGGAAGATGCCAAAAAC-3′ including NheI restriction site (underlined) and Kozac sequence (bold) and reverse 5′- *GCGGCCGC*GGATCCCACGGCGATCTTGCCGCC-3′ including NotI restriction site (underlined). PCR product and pCDH.MCS.T2A.Puro-MSCV were both digested with NheI and NotI and ligated by using T4 DNA ligase. The final pMSCV.luc2.T2A.Puro plasmid was sequence-verified. Pseudo-typed lentiviral particles encoding Luc2 reporter and puromycin resistance genes were produced in HEK 293 T cells by co-transfection with accessory vectors pΔ8.9 and pMD.G by using *FuGene 6* transfection reagent (Roche) in accordance with the guidelines of the manufacturer.

After a 24-hour incubation, culture media were replaced with DMEM-HG supplemented with 10% FBS. Forty-eight and 72 hours after transfection, culture media containing lentiviral particles were filtered (0.45 μm; Corning Life Sciences, Tewksbury MA, USA) and concentrated by ultracentrifugation at 45,000 *g* for 2 hours at 4°C.

### Transduction and cp-MSCs selection

cp-MSCs were transduced in the presence of polybrene (8 μg/mL; Sigma-Aldrich). After 24 hours, culture medium was replaced with the standard expansion medium. Approximately 48 hours after transduction, 0.2 μg/mL of puromycin was added to the medium and changed every other day. Cells were selected during 7 days and then expanded for bioluminescence imaging assay.

### Bioluminescence imaging assay

For *in vitro* studies, transduced cells were plated in a 24-well plate at different concentrations: 10^4^, 2 × 10^4^, 4 × 10^4^, 6 × 10^4^, 8 × 10^4^, and 10^5^ cells per well. D-Luciferin (150 μg/mL) (Promega Corporation) was added to the culture medium in accordance with the guidelines of the manufacturer. The culture plate was immediately positioned in the IVIS Lumina Imaging System (Caliper Life Sciences, Hopkinton, MA, USA), and images were acquired after a 10-second exposure period.

For *in vivo* studies, mice received D-Luciferin (150 mg/kg) intraperitoneally. Ten minutes after injection, they were anesthetized with isoflurane gas and placed in the IVIS Lumina Imaging System. Image acquisitions were performed 1, 2, 3, and 5 days after the injection of transduced cells. Exposure time never exceeded 5 minutes, to avoid false-negative results. *In vitro* and *in vivo* results were analyzed with Living Image Software 3.2 software (Caliper Life Sciences).

### Histology

At 40 days after the first injection, mice were sacrificed and hearts were perfused with paraformaldehyde 4%. Cardiac tissue was embedded in paraffin, and 5-μm sections were used to quantify the fibrotic scar by using Sirius Red staining. Three measurements were made by using sections from the apex toward the base of the heart, and these values were averaged. Infarct area was estimated by morphometry as a percentage of the total area of the left ventricle by using Image-Pro Plus 7.0.1 software.

### Quantitative reverse transcription-polymerase chain reaction

Total RNA was isolated by using RNeasy Plus Mini kit (Qiagen, Valencia, CA, USA) in accordance with the instructions of the manufacturer. Total RNA (500 ng) was used for cDNA synthesis by using a High Capacity cDNA Reverse Transcription kit (Applied Biosciences) in accordance with the instructions of the manufacturer. The Human Cell Cycle RT^2^ profiler PCR array (PAHS-020; SABiosciences, part of Qiagen) was used to analyze the expression of genes related to cell cycle regulation in cp-MSCs and cv-MSCs. The expression of POU5F1 was also evaluated in these cells in a different set of experiments. The Mouse Insulin Signaling Pathway RT^2^ Profiler PCR array (PAMM-030; SABiosciences) was used to compare gene expression in placebo- and cell-treated mouse hearts. A complete list of the genes analyzed is presented in supplementary tables (Additional files 3 and 4). The relative quantities of gene-specific mRNAs were calculated by using the 2^-(∆∆Ct)^ method and web-based software available at the SAbiosciences website.

### Statistical analysis

All values are expressed as mean ± standard error. Cardiac function parameters were compared between groups by using repeated measures one-way analysis of variance (ANOVA) and, for time-dependent analysis, two-way ANOVA. Comparisons between two groups were performed by using the Student *t* test. Differences between variables were considered significant when the *P* value was not more than 0.05. The GraphPad Prism^®^ software, version 5.0 (GraphPad Software, Inc., La Jolla, CA, USA), was used for all statistical analyses.

## Results

### Placenta-derived MSC morphology, surface phenotype, and differentiation

Two days after initial plating, both cp-MSCs and cv-MSCs showed adherence to plastic and displayed fibroblast-like morphology (n = 16), which was maintained for 15 passages (data not shown). Third-passage cells can be seen in Additional file 5A and B. Primary cultures also contained non-adherent cells, which were eliminated through media change. Adherent, fibroblast-like cells derived from chorionic plate and chorionic villi in the primary culture reached confluence in approximately 4 and 8 days, respectively.

**Figure 1:**
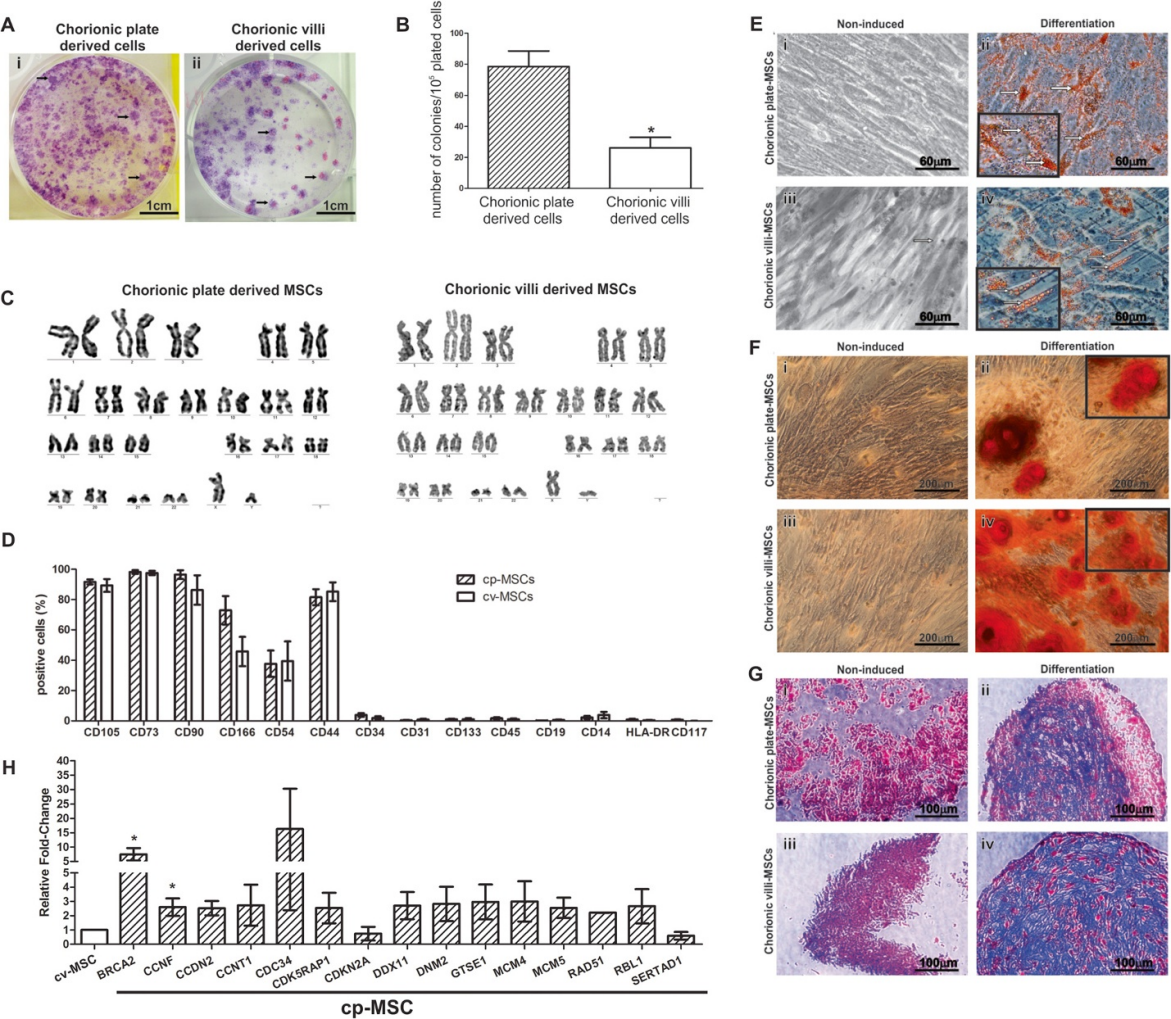
**Characterization of placenta-derived cells. (A)** Fibroblast colony-forming units (CFU-F) assay. Freshly isolated cells derived from (i) chorionic plate (cp) and (ii) chorionic villi (cv) were cultured in CFU-F forming conditions and stained with Giemsa. Black arrows indicate colonies. **(B)** The quantity of clones formed by cp-derived cells (78.5 ± 10.1) and cv-derived cells (31.4 ± 5.2) was compared (*P = 0.0015). **(C)** Karyotype analysis of cp- and cv-mesenchymal stem cells (MSCs) showing a 46, XY karyotype in both cases. **(D)** Flow cytometry analysis. Graph shows the percentage of positive cells for each surface marker in cp-MSCs (striped) and cv-MSCs (white). Expression of mesenchymal markers and adhesion molecules was high, while low expression of hematopoietic and endothelial markers was found. **(E)** Oil Red O staining after adipogenic differentiation. Cytoplasmic lipid droplets were observed in the induced (ii) cp-MSCs and (iv) cv-MSCs but were not present in the non-induced cultures (i and iii). White arrows indicate lipid droplets within the cytoplasm. **(F)** Alizarin Red staining after osteogenic differentiation. Calcium deposits in the extracellular matrix were observed in the induced (ii) cp-MSCs and (iv) cv-MSCs but were not present in the non-induced cultures (i and iii). Inserts in (E) and (F) show images in higher magnification. **(G)** Analysis of 3-week cultured pellets after chondrogenic differentiation. The micromass was stained with Alcian Blue. Nuclei were counterstained with Nuclear Fast Red. Proteoglycans, stained in blue, were observed in the induced (ii) cp-MSCs and (iv) cv-MSCs. Non-induced cultures (i and iii) did not present proteoglycan staining. **(H)** Quantitative reverse transcription-polymerase chain reaction (RT-PCR) array for cell cycle-related genes. Results are shown as fold changes in expression of cp-MSCs (stripped bars) when compared with cv-MSCs (white bar). All genes that were at least twofold up- or downregulated in cp-MSCs when compared with cv-MSCs are shown. Expression of BRCA2 and CCNF was significantly higher in cp-MSCs (*P <0.05).

We used flow cytometry to investigate the surface phenotype of placenta-derived cells (Additional file 6). It revealed that cp-MSCs (n = 5) and cv-MSCs (n = 4) were positive for MSC-related markers CD90, CD73, and CD105 as well as adhesion molecules CD54, CD44, and CD166. Moreover, they showed very low expression of hematopoietic surface markers, such as CD45, CD34, CD117, CD14, CD19, and HLA-DR and endothelial markers CD133 and CD31. The phenotype observed in cp-MSCs and cv-MSCs was consistent with the profile of human bone marrow-derived MSCs. We found no significant differences in the expression of mesenchymal, endothelial, or hematopoietic molecules between cp-MSCs and cv-MSCs (Figure 1D).To evaluate multipotency of cp-MSCs and cv-MSCs, we differentiated them into adipocytes, osteoblasts, and chondroblasts. After adipogenic differentiation, cp-MSCs (Figure 1Eii) and cv-MSCs (Figure 1Eiv) accumulated lipid-rich vacuoles, stained in red by Oil Red O. Control cultures were maintained with expansion medium and, at the end of protocol, did not show lipid accumulation (Figure 1Ei and iii). After osteogenic differentiation, cp-MSCs (Figure 1Fii) and cv-MSCs (Figure 1Fiv) showed calcium deposits in the extracellular matrix, stained in red by Alizarin Red, which characterizes matrix mineralization. Moreover, no spontaneous differentiation was observed in control cultures (Figure 1Fi and iii).When cp-MSCs and cv-MSCs were cultured in micromass-forming conditions, the formation of a single aggregate was observed after 24 hours in both induced and non-induced cells. After Alcian Blue and Nuclear Fast Red staining, paraffin sections of induced cp-MSCs (Figure 1Gii) and cv-MSCs (Figure 1Giv) showed synthesis of proteoglycans in blue, which are characteristic of the cartilaginous matrix, and nuclear staining in red. Paraffin sections of non-induced cp-MSCs and cv-MSCs showed no proteoglycan staining (Figures 1Gi and iii).

### Clonogenic properties, growth kinetics, and chromosomal stability of placenta-derived MSCs

To analyze cell clonogenic potential, CFU-F assays were performed in single-cell suspensions from chorionic plate (n = 6) and chorionic villi (n = 5) preparations. Both of them were able to generate CFU-F (Figure 1A). Colonies stained with Giemsa solution were quantified. Chorionic plate- and chorionic villi-derived cells yielded 78.5 ± 10.1 and 31.4 ± 5.2 colonies per 10^5^ plated cells, respectively (*P* = 0.0015) (Figure 1B). Thus, cp-MSCs were at least two times more efficient in generating clones than cv-MSCs.

When cultured in expansion medium, third-passage cp-MSCs and cv-MSCs plated in 35-mm dishes for PDT assay reached 100% confluence in approximately 8 days. With daily monitoring of cellular growth, we were able to show that the proliferation rate of both cell types followed an exponential curve (Additional file 7A). The time required to double the cell population was estimated by calculating the inverse of the angular coefficient obtained from the linear regression formed after applying a base 2 logarithm in the cells per millimeter squared axis (Additional file 7B). No statistical difference was observed between cp-MSCs and cv-MSCs PDT (1.7 ± 0.1 *versus* 2.3 ± 0.5, respectively; *P* = 0.4) (Additional file 7C). However, there is a clear tendency toward a faster growth of cp-MSCs compared with cv-MSCs.Karyotype analysis was performed on GTG-banded metaphases from cp-MSCs (n = 3) and cv-MSCs (n = 3). All 20 metaphases that were analyzed from each sample were normal (karyotype: 46, XY) (Figure 1C).

In addition, expression of isoform 1 of the POU5F1 gene was detected in both cell types (Additional file 8) after excluding the artifactual detection of the POU5F1B gene or the presence of pseudogenes (Additional file 8C). Nonetheless, levels of POU5F1 were much lower in placenta-derived cells than in human embryonic stem cells (data not shown).

### Distinct transcript profile of cell cycle-related genes in cv- and cp-MSCs

Quantitative reverse transcription-polymerase chain reaction arrays were used to measure differences in the transcript levels of genes related to cell cycle regulation. We identified a difference of at least twofold in the expression of 15 cell cycle-related genes (Figures 1H and Additional file 9). Importantly, there was a significant difference in the expression of BRCA2 (6.85-fold, *P* = 0.02), which is critical for the control of homologous recombination and DNA damage repair, and CCNF (2.42-fold, *P* = 0.03) in cp-MSCs when compared with cv-MSCs.

### Myocardial infarction and therapy with cp-MSCs

Since cp-MSCs had a greater clonogenic potential and expressed more genes related to cell cycle progression and stability, we considered them a preferable source for cell therapy in our model. At 20 days after MI, before cell or placebo treatment, cp-MSCs and placebo groups presented a significant decrease in EF and increase in ESV and EDV when compared with the sham group (Table 1). No statistical difference was observed in cardiac function between mice in the cp-MSCs or placebo group at this time point (Table 1).

**Table 1:** **Cardiac function of experimental groups**

		Experimental groups		
Parameters	Time, days ^a^	Sham (n = 11)	cp-MSCs (n = 11)	Placebo (n = 8)
Ejection fraction, %	-22	57.5 ± 3.6	59.5 ± 5.6	59.3 ± 3.6
	-1	50.2 ± 1.7	28.7 ± 2.1^b^	27.4 ± 2.6^b^
	7	49.8 ± 2.4	34.3 ± 3.1	26.4 ± 3.0
	15	50.9 ± 2.6	33.8 ± 1.9	22.9 ± 2.4^c^
	23	48.3 ± 1.8	31.2 ± 3.1	24.6 ± 2.6
	39	46.2 ± 2.6	33.8 ± 2.3	22.2 ± 2.1^d^
End-systolic volume, μL	-22	24.4 ± 3.9	23.3 ± 7.4	17.7 ± 3.8
	-1	31.3 ± 2.3	61.4 ± 4.7^b^	67.2 ± 5.5^b^
	7	31.6 ± 2.7	55.5 ± 6.2	62.4 ± 7.3
	15	34.35 ± 3.4	60.4 ± 5.3	75.8 ± 6.8^c^
	23	34.5 ± 2.2	64.6 ± 6.5	77.2 ± 6.4
	39	34.7 ± 3.5	58.3 ± 7.9	80.7 ± 8.5^c^
End-diastolic volume, μL	-22	56.3 ± 4,9	54.8 ± 9.5	53.8 ± 8.5
	-1	62.3 ± 3.4	84.2 ± 4.3^b^	92.0 ± 4.8^b^
	7	62.6 ± 4.2	82.5 ± 5.6	90.9 ± 7.9
	15	68.6 ± 4.2	90.6 ± 6.9	98.0 ± 6.9
	23	67.2 ± 3.9	91.8 ± 6.1	102.6 ± 7.3
	39	63.6 ± 4.2	89.9 ± 9.2	104.3 ± 9.6

^a^Day -22 shows baseline values (before myocardial infarction) for all groups. Day -1 shows data before treatment with either chorionic plate mesenchymal stem cells (cp-MSCs) or placebo. ^b^ *P* <0.05 compared with Sham. ^c^ *P* <0.05, ^d^ *P* <0.01 comparing cp-MSCs *versus* placebo.

**Figure 2:**
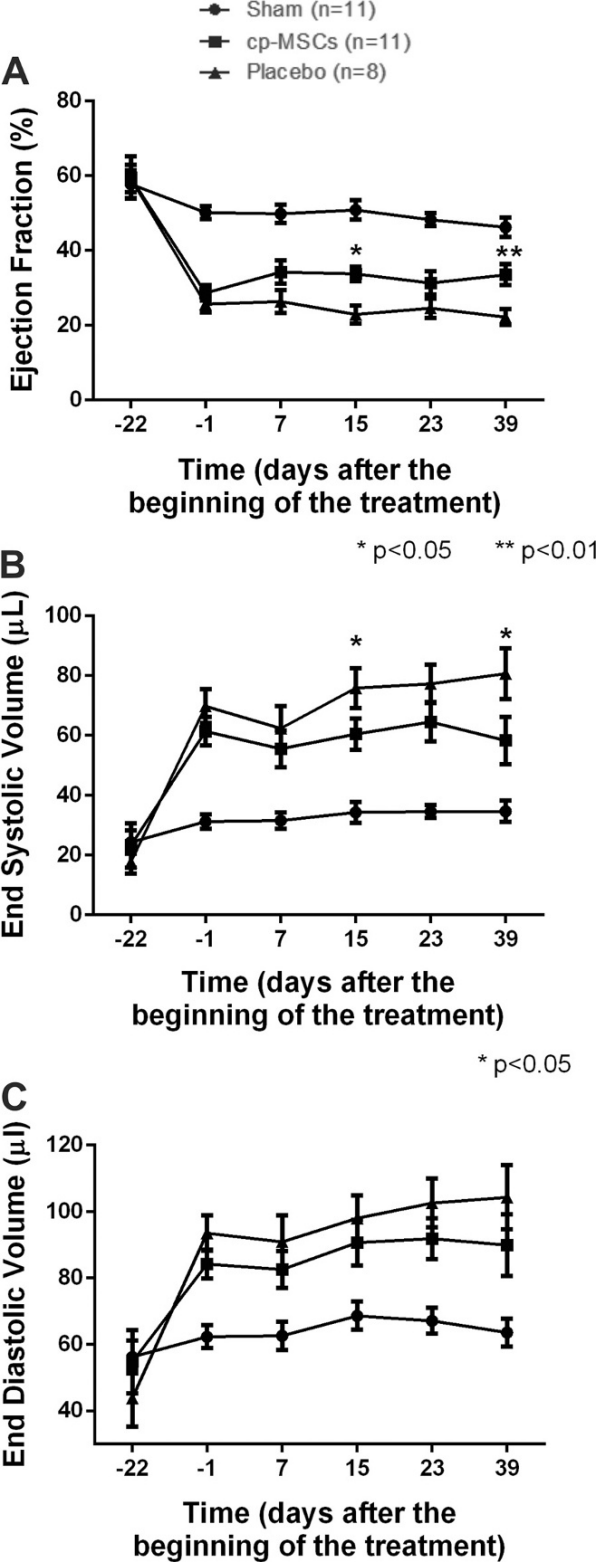
**Assessment of cardiac function in infarcted mouse hearts transplanted with chorionic plate mesenchymal stem cells (cp-MSCs) or placebo.** Echocardiography was performed before (corresponding to day -22 of treatment) and 20 days after myocardial infarction (corresponding to day -1 of treatment) to obtain baseline values in mice receiving cp-MSCs (squares) or placebo (triangles). Analysis of **(A)** ejection fraction, **(B)** end-diastolic volume, and **(C)** end-systolic volume was performed every 7 days after each cell or placebo injection. Day 7 corresponds to 7 days after the first injection, day 15 corresponds to 7 days after the second injection, and day 23 corresponds to 7 days after the third injection. Animals were followed up to 39 days after the first injection. *P <0.05 and **P <0.01 when comparing cp-MSC with placebo group at each time point. Sham-operated mice (circles), which did not undergo left anterior descending coronary artery occlusion, are shown as controls to indicate normal values for each parameter.

EF and ESV were significantly improved in the cp-MSC group when compared with placebo at 15 and 39 days after cell or vehicle injection (Table 1 and Figure 2A and C). Cell therapy had no significant effect on EDV when comparing cell-treated and placebo groups (Table 1 and Figure 2B).

### Determination of cell engraftment by bioluminescence

cp-MSCs were transduced with a lentiviral vector containing the luciferase 2 and puromycin resistance genes under the control of MSCV promoter. Imaging of a dilution series containing 1 to 10 × 10^4^ cells *in vitro* showed a strong linear correlation between photon emission and cell number (R^2^ = 0.95) (Additional file 10A and B), indicating that bioluminescence offers a reliable estimation of cell numbers.

**Figure 3:**
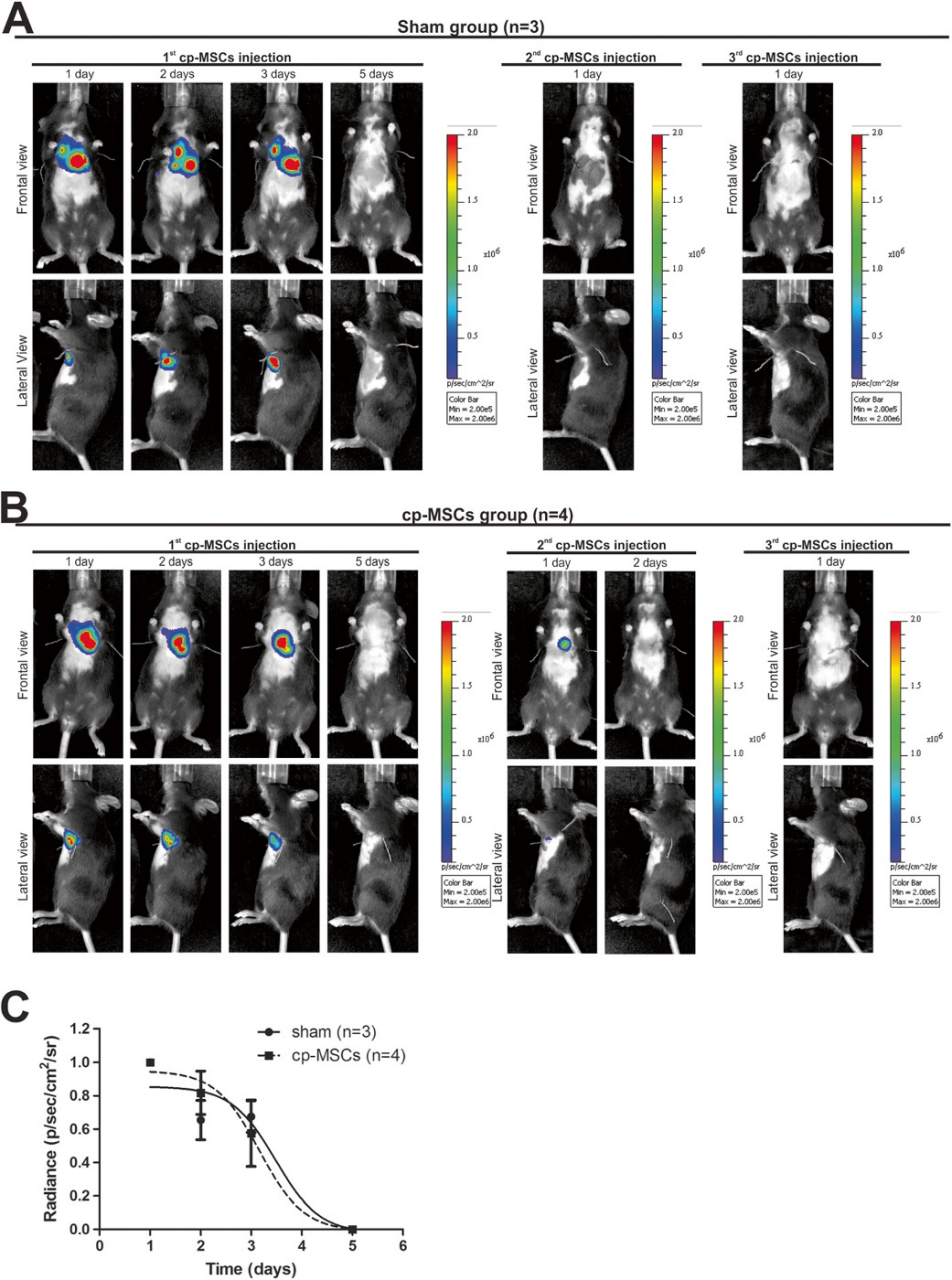
***In vivo*** **imaging of transduced chorionic plate mesenchymal stem cell (cp-MSCs) administered by intramyocardial injection in sham and infarcted mice.** Bioluminescence imaging was repeated daily. After the first injection, the signal was located in a region anatomically compatible with the heart in sham **(A)** and infarcted **(B)** mice injected with cp-MSCs. After the second and third injections, the luminescent signal was observed for only 1 or 2 days in these animals. In addition, the signal intensity was lower than after the first injection. The scales represent luminescence intensity in radiance units, in which red and blue are the most and least intense, respectively. **(C)** Signal quantification in sham (circles) and infarcted (squares) groups after the first injection of cp-MSCs. Quantification of the bioluminescent signal showed no statistical difference in the engraftment period for both groups.

To determine the distribution of Luc2-transduced cp-MSCs after intramyocardial injection in sham (n = 3) or infarcted (n = 4) mice, bioluminescence imaging was performed 10 minutes after intraperitoneal administration of D-Luciferin. Luciferase expression was monitored *in vivo* by real-time imaging of the whole body, and this signal was superimposed to a light photograph to provide an anatomical reference. Repeated analyses on the same animal at different time points allowed the study of the biodistribution and engraftment of transduced cp-MSCs in healthy and infarcted hearts.In sham and infarcted mice, cells were detected in only the thoracic region. In both groups, after the first cp-MSCs injection, most of the signal was lost after the third day, suggesting that cells did not engraft permanently in the heart (Figure 3). For quantification purposes, the signal in the following days was normalized to the signal at 24 hours after injection. No statistical difference was observed between sham and infarcted animals (Figure 3C). Moreover, after the second and third cp-MSCs injections, cells remained for even less time in the heart since the signal was predominantly absent only 24 hours after injection in both sham and infarcted mice (Figure 3). These data indicate that permanent engraftment is not required for the improvement of cardiac function in cp-MSC therapy.

### Quantification of infarct area

**Figure 4:**
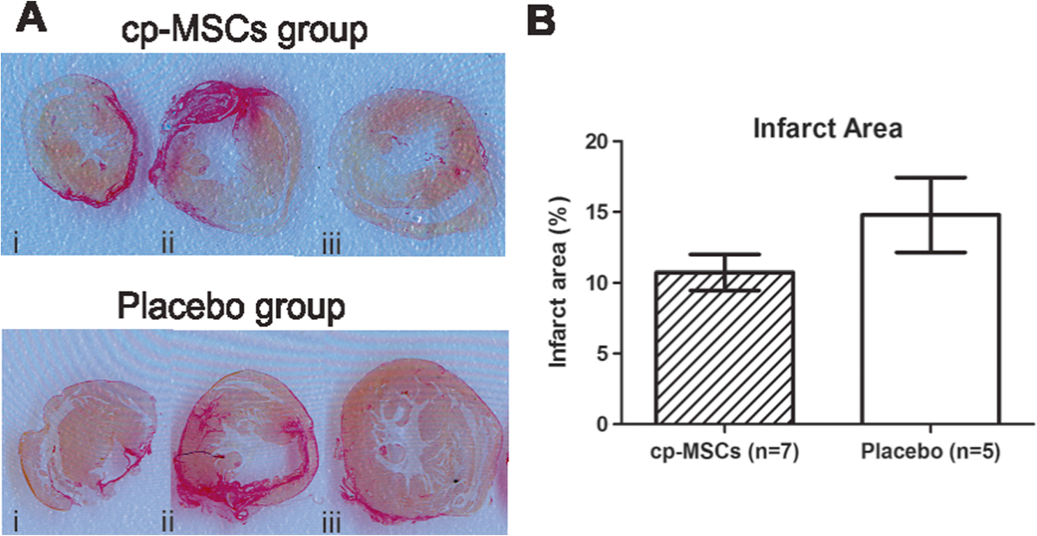
**Impact of chorionic plate mesenchymal stem cell (cp-MSC) injection on infarct area. (A)** Representative example of scar formation 39 days after the first injection in mice treated with cp-MSCs or placebo. Macroscopic infarct area was assessed in each group by Sirius Red staining. Fibrous scar tissue is shown in red, whereas myocardium is stained in yellow. Serial sections of the same heart are shown from apex (i) to base (iii). **(B)** Infarct area (red-stained tissue) was quantified as a percentage of total area. The graph shows an average of infarct area calculated from the three serial sections. No statistical difference was observed in the scar formation between cp-MSC and placebo groups.

The absence of cell engraftment in bioluminescence experiments suggested that direct regeneration of cardiac muscle was likely not involved in the improvement of function. The fact that no difference was found between infarct areas in placebo- and cell-treated groups further supports this notion. Sirius red staining showed that 14.8% ± 2.6% and 10.7% ± 1.2% of the left ventricle were infarcted in the placebo and cp-MSCs groups, respectively (Figure 4).

### Insulin signaling pathway regulation

Transcript levels of genes associated with the insulin signaling pathway were evaluated 39 days after therapy in the heart of mice treated with either cp-MSCs (n = 3) or placebo (n = 3). No differences were found in the majority of the transcripts analyzed in these hearts (Additional file 11). Among those transcripts in which statistical differences were found between cell-treated and placebo groups, none of them had a variation of more than twofold, suggesting that these results might not be biologically relevant (Additional file 11).

## Discussion

The application of MSCs in cell therapy and regenerative medicine is promising, and their beneficial effects have been demonstrated in several murine disease models, such as stroke [39], chronic airway inflammation [40], and acute MI [41–44]. However, to the best of our knowledge, the effects of human placenta MSC transplantation have not been evaluated in a murine model of healed MI.

Our first objective was to determine which would be the preferable source of placenta MSCs. Since these cells can be isolated from two different regions, the chorionic plate [23–26] or the chorionic villi [13–15, 20], a full characterization of MSCs derived from these two regions was performed. At first, one might think that cp-MSCs and cv-MSCs are equivalent given that the chorionic villi arise from the chorionic plate during human development. Even though these cells are quite similar in several aspects, the difference in their clonogenic potential seemed important to us. Since the capacity of generating clones is related to the amount of stem cells in the starting sample, our results suggest that stem cells are present in higher numbers in the chorionic plate.

In addition, key cell cycle regulators were also differentially expressed in cp-MSCs. BRCA2 is a positive regulator of homologous recombination through its interaction with RAD51 recombinase [45]. It acts recruiting RAD51 to double-strand breaks and initiating DNA repair, which is crucial for the maintenance of genome integrity [45]. Importantly, RAD51 was increased 2.22-fold in cp-MSCs, although this result did not reach statistical significance. Cyclin F (CCNF) was also significantly upregulated in cp-MSCs. This molecule drives cell cycle progression and also plays a role in DNA repair and genome integrity [46]. Moreover, cyclin F seems to be particularly important for the placenta since its deletion is lethal during embryonic development because of failure in yolk sac and chorioallantoic placentation [47].

Given their superior clonogenic potential and increased expression of genome integrity regulators, we selected cp-MSCs as our candidates for cell therapy in a murine model of MI. In accordance with a recent statement from the American Heart Association [48], our model fulfilled functional criteria of dilated cardiomyopathy and heart failure, as demonstrated by the increase in EDV and reduction in EF before treatment with cp-MSCs. Therapy was given after MI was completely healed by using three weekly intramyocardial injections of either cp-MSCs or placebo. Evidence from other disease models demonstrates that repeated injections of MSCs are superior to a single injection [49, 50], which is expected in any therapeutic approach. The difficulty of performing repeated injections by using an intramyocardial delivery route is the need for repeated thoracotomies, which increase mortality substantially. To overcome this issue, our cell delivery procedure did not require thoracotomy since it was guided by echocardiography and cp-MSCs were successfully injected, as demonstrated by the bioluminescence data. Forty days after the first injection, cell therapy with cp-MSCs led to an improvement in systolic function, since ESV was reduced and EF was increased, but did not influence cardiac remodeling, as dilation was not diminished.

To investigate whether the cells remained in the myocardium after injection, we performed cell tracking experiments by bioluminescence. We considered this to be an ideal approach since our data demonstrated a strong correlation between the bioluminescent signal and the amount of cells. In addition, since luciferin metabolism requires ATP, only living cells expressing luciferase are capable of producing signal. Our data demonstrated that the presence of injury did not influence the amount of time that cp-MSCs remained in cardiac tissue and that engraftment was not permanent. In fact, cells disappeared quite rapidly and did not migrate to other organs. Moreover, cells remained even less time in the tissue after the second and third injections. Engraftment has become a critical issue for cell-based therapies as many groups have demonstrated that the majority of the injected cells are lost after very few days [51]. In our model, one could argue that cell loss could be due simply to immune rejection of cp-MSCs in a xenogeneic transplantation setting, especially after the second and third injections. There are some points that need to be made in this regard. The immunomodulatory properties of MSCs are well known [1, 52], even though most *in vivo* models focus on allogeneic, not xenogeneic, scenarios and, in the case of human MSCs, on *in vitro*, rather than *in vivo*, experiments. However, a number of articles in different disease models show that xenotransplantation of MSCs has a therapeutic effect (for a review on this subject, see [53]). Lastly, the fact is that, in a syngeneic, allogeneic, or xenogeneic setting, permanent engraftment of transplanted cells does not occur [51, 54]. The absence of bioluminescent signal indicates that few cells, if any, remained in the myocardium, suggesting that regeneration due to transdifferentiation of cp-MSCs was not the primary mechanism involved in the improvement of cardiac function and favoring the “paracrine hypothesis”.

The paracrine hypothesis proposes that the benefits promoted by cell therapy are due to the release of soluble factors that induce angiogenesis and exert cardioprotective effects [55, 56]. The insulin signaling pathway has been implicated as an important contributor to this effect [55, 57]. However, our results show that the effect of MSC therapy does not seem to be mediated by the insulin signaling pathway, as we did not find substantial changes in genes related to this pathway when comparing cell- and placebo-treated hearts. Nonetheless, since our analysis was performed 39 days after treatment, we cannot exclude that this pathway was modulated at a different time point. In addition, the number of analyzed animals in each group was small (n = 3), and although quantitative PCR arrays are very sensitive, subtle differences in gene expression could be masked.

One of the major issues in recognizing that the mechanism of action using MSCs might be paracrine resides in the fact that one could simply administer soluble factors and reach the same result [55]. However, aside from the many caveats of this type of therapy, such as protein stability and pharmacokinetics, soluble factors lack a property that we consider to be crucial: a sensor system that responds and is modulated by the environment. Being “sensitive” is a unique property of cell-based approaches and, although it may be one of its strongest suits, it is also responsible for its mechanistic elusiveness. The same cell might respond in several different ways, depending on the stimuli it receives from a given tissue; thus, we might never be able to fully understand the crosstalk that takes place in this scenario. On the other hand, despite the mechanistic discussion that has been dominating the field in basic research [58, 59], cell therapy with MSCs has proven to be safe in the clinical setting [52, 60], and data of phase II trials in cardiovascular diseases should become available in the next years.

## Conclusions

We have extensively characterized MSCs derived from the two placenta regions commonly used for cell isolation, showing many similarities but some important differences that point to the chorionic plate as a preferable source for placenta MSCs. Moreover, we demonstrate that cp-MSCs improve cardiac function after MI, an effect that was not dependent on permanent cell engraftment and did not involve modulation of the insulin signaling pathway. Hence, the placenta is a suitable source of MSCs for allogeneic cell therapy and has the further advantage of being a virtually inexhaustible tissue that can be easily obtained without invasive procedures and cell-banked.

## Electronic supplementary material

Additional file 1: **Methods of isolation for mesenchymal stem cells (MSCs) from different regions of the human term placenta** **[** 13 **-** 20 **,** 22 **,** 23 **,** 25 **,** 26 **,** 61 **-** 66 **].** (DOCX 27 KB)

Additional file 2: **Experimental protocol.** Numbers represent days before or after the first injection of chorionic plate mesenchymal stem cells (cp-MSCs) or placebo (therapy at day 0). ECG, electrocardiogram; ECHO, echocardiogram; Histo, histology. (TIFF 102 KB)

Additional file 3: **Genes analyzed by the cell cycle pathway polymerase chain reaction (PCR) array.** (DOCX 119 KB)

Additional file 4: **Genes analyzed by the insulin signaling pathway polymerase chain reaction (PCR) array.** (DOCX 119 KB)

Additional file 5: **Morphology of placenta-derived mesenchymal stem cells (MSCs) in phase contrast microscopy.** Third-passage MSCs from **(A)** chorionic plate and **(B)** chorionic villi presented fibroblast-like cell morphology. (TIFF 807 KB)

Additional file 6: **Flow cytometry analysis.** Representative flow cytometry histograms of surface molecule expression in chorionic plate mesenchymal stem cells (cp-MSCs) **(A)** and chorionic villi mesenchymal stem cells (cv-MSCs) **(B)**. The fluorescence intensity for each molecule is shown in the x-axis. Isotype controls are represented by the light gray curve. Positive events were calculated by subtracting the events obtained using the primary antibody from the isotype control. The average percentage of positive events ± standard error of the mean (SEM) is shown in the upper right corner of each histogram; 7 aminoactinomyocin D (7AAD) (green) was used to exclude dead cells. (TIFF 1 MB)

Additional file 7: **Population doubling time (PDT) assay. (A)** Daily quantification of cp-MSCs (circles and full line) and cv-MSCs (squares and dashed line) showed an exponential growth. **(B)** The exponential curves shown in (A) were transformed by using a log2 scale in the y-axis. PDT was calculated by performing linear regression and using the inverse of the slope (or angular coefficient) as an estimate of duplication time. **(C)** PDT values in days from independent experiments are shown for cp-MSCs and cv-MSCs. Above the bars, mean ± standard error of the mean (SEM) values of PDT are indicated for each cell type. (TIFF 206 KB)

Additional file 8: **Expression of POU5F1 (NM_002701.4) in placenta-derived cells. (A)** Reverse transcription-polymerase chain reaction (RT-PCR) detection of transcripts POU5F1 (136 bp) and GAPDH (162 bp) in chorionic plate mesenchymal stem cells (cp-MSCs) (lanes 1 and 3), chorionic villi mesenchymal stem cells (cv-MSCs) (lanes 2 and 4), and human embryonic stem cells (lane 5). Samples in lanes 1 and 2 were derived from chorionic plate and chorionic villi obtained from the same placenta. The same is true for samples in lanes 3 and 4. **(B)** Since the expression of POU5F1 in adult MSCs is controversial [67, 68], we designed primers that recognize transcript variant 1 of POU5F1 but that do not recognize transcript variants 2 and 3, which are not expressed in pluripotent stem cells. Moreover, to differentiate POU5F1 from POU5F1B (NM_001159542.1), which is a different gene not related to pluripotency, both primers have a mismatch in the last nucleotide (underlined), which prevents amplification of POU5F1B. **(C)** To further confirm our results, PCR products were sequenced and compared with POU5F1 transcript variant 1, POU5F1B, and pseudogenes 3 and 4. Light gray bases show similarities between sequences. Black bases represent mismatches. The PCR product sequence (28V_F) shows 100% similarity only to POU5F1 transcript variant 1. Thus, sequence alignment analysis revealed that adult MSCs express transcript variant 1 of POU5F1. Nevertheless, it is likely that other transcript variants (2 and 3), POU5F1B and/or pseudogenes are also expressed. We immunostained placenta-derived MSCs and detected the presence of nuclear OCT4 protein (data not shown). However, OCT4 (product of POU5F1) has 96% homology to OCT4B (product of POU5F1B) [68], making it impossible to discriminate between them with commercially available antibodies. Finally, it is difficult to speculate which function POU5F1 might have in these cells since they are not pluripotent. (TIFF 2 MB)

Additional file 9: **Expression of cell cycle-related genes in chorionic plate mesenchymal stem cells (cp-MSCs) compared with chorionic villi mesenchymal stem cells (cv-MSCs). (A)** Heat map shows log2 (fold-change) of downregulated (green) and upregulated (red) genes in cp-MSCs when compared with cv-MSCs. **(B)** Table identifies and specifies mean fold-change values for each of the genes analyzed by quantitative reverse transcription-polymerase chain reaction (qRT-PCR). Targets with at least twofold upregulation are shown in red, whereas targets with at least twofold downregulation are shown in blue. Full names and accession numbers for all the genes can be found in Additional file 3. (TIFF 822 KB)

Additional file 10: **Luciferase 2 expression in transduced chorionic plate mesenchymal stem cells (cp-MSCs). (A)** Bioluminescence imaging of cp-MSCs *in vitro* shows higher luminescent signal with the increase of cell density. On the right, a scale of the luminescent signal is shown using radiance units. **(B)** Graph shows a linear correlation between cell numbers and the emitted radiance (R^2^ = 0.95). (TIFF 542 KB)

Additional file 11: **Expression of insulin signaling pathway-related genes in the hearts of chorionic plate mesenchymal stem cell (cpMSC)-treated compared with placebo-treated mice. (A)** Heat map shows log2 (fold-change) of downregulated (green) and upregulated (red) genes in cp-MSCs when compared with placebo-treated mice. **(B)** Table identifies and specifies mean fold-change values for each of the genes analyzed by quantitative reverse transcription-polymerase chain reaction (qRT-PCR). The vast majority of the genes had less than twofold difference between experimental groups. The four cases with more than twofold difference (Cfd, Gck, Irs2, and Nos2) did not reach statistical significance. Full names and accession numbers for all the genes can be found in Additional file 4. (TIFF 1 MB)

## Abbreviations

ANOVA: analysis of variance
CFU-F: fibroblast colony-forming units
cp-MSC: chorionic plate mesenchymal stem cell
cv-MSC: chorionic villi mesenchymal stem cell
EDV: end-diastolic volume
EF: ejection fraction
ESV: end-systolic volume
FBS: fetal bovine serum
LAD: left anterior descending coronary artery
Luc2: luciferase 2
MI: myocardial infarction
MSC: mesenchymal stem cell
PBS: phosphate-buffered saline
PCR: polymerase chain reaction
PDT: population doubling time.
