# Supplementary material for: Improvement of cardiac function by placenta-derived mesenchymal stem cells does not require permanent engraftment and is independent of the insulin signaling pathway
Source: Stem Cell Res Ther. 2014 Aug 21;5(4):102. doi: 10.1186/scrt490 (PMC4354978; doi:10.1186/scrt490)
Supplement: Supplementary file 1 — Additional file 1: Methods of isolation for mesenchymal stem cells (MSCs) from different regions of the human term placenta [ 13 - 20 , 22 , 23 , 25 , 26 , 61 - 66 ]. (DOCX 27 KB) [file 13287_2014_412_MOESM1_ESM.docx]

Additional File 1. Methods of isolation for MSCs from different regions of the human term placenta.

| **Isolation method** | **Stem cell source** | **Reference** |
| --- | --- | --- |
| Collagenase plus dispase II | Chorionic plate | 22 |
|  | Chorionic villi | 22  16 |
| Collagenase plus  DNAse I | Chorionic plate | 26 |
| Collagenase | Chorionic villi | 14 |
| Trypsin plus EDTA | Chorionic villi | 13  15  17  20  6[1](#_ENREF_3) |
|  | Chorionic plate | 6[2](#_ENREF_4)  6[1](#_ENREF_3) |
|  | Whole placenta | 6[3](#_ENREF_5)  6[4](#_ENREF_6)  6[5](#_ENREF_7) |
| Collagenase plus Trypsin | Whole placenta | 6[6](#_ENREF_8) |
| Mechanical mincing | Chorionic villi | 18  19 |
|  | Chorionic plate | 23  25 |
