# Supplementary material for: Improvement of cardiac function by placenta-derived mesenchymal stem cells does not require permanent engraftment and is independent of the insulin signaling pathway
Source: Stem Cell Res Ther. 2014 Aug 21;5(4):102. doi: 10.1186/scrt490 (PMC4354978; doi:10.1186/scrt490)
Supplement: Supplementary file 3 — Additional file 3: Genes analyzed by the cell cycle pathway polymerase chain reaction (PCR) array. (DOCX 119 KB) [file 13287_2014_412_MOESM3_ESM.docx]

Additional File 3. Genes analyzed by the cell cycle pathway PCR array.

| **Symbol** | **Description** | **Accession number** |
| --- | --- | --- |
| ABL1 | C-abl oncogene 1, non-receptor tyrosine kinase | NM_005157 |
| ANAPC2 | Anaphase promoting complex subunit 2 | NM_013366 |
| ANAPC4 | Anaphase promoting complex subunit 4 | NM_013367 |
| DIRAS3 | DIRAS family, GTP-binding RAS-like 3 | NM_004675 |
| ATM | Ataxia telangiectasia mutated | NM_000051 |
| ATR | Ataxia telangiectasia and Rad3 related | NM_001184 |
| BAX | BCL2-associated X protein | NM_004324 |
| BCCIP | BRCA2 and CDKN1A interacting protein | NM_016567 |
| BCL2 | B-cell CLL/lymphoma 2 | NM_000633 |
| BIRC5 | Baculoviral IAP repeat containing 5 | NM_001168 |
| BRCA1 | Breast cancer 1, early onset | NM_007294 |
| BRCA2 | Breast cancer 2, early onset | NM_000059 |
| CCNB1 | Cyclin B1 | NM_031966 |
| CCNB2 | Cyclin B2 | NM_004701 |
| CCNC | Cyclin C | NM_005190 |
| CCND1 | Cyclin D1 | NM_053056 |
| CCND2 | Cyclin D2 | NM_001759 |
| CCNE1 | Cyclin E1 | NM_001238 |
| CCNF | Cyclin F | NM_001761 |
| CCNG1 | Cyclin G1 | NM_004060 |
| CCNG2 | Cyclin G2 | NM_004354 |
| CCNH | Cyclin H | NM_001239 |
| CCNT1 | Cyclin T1 | NM_001240 |
| CCNT2 | Cyclin T2 | NM_001241 |
| CDC16 | Cell division cycle 16 homolog (S. cerevisiae) | NM_003903 |
| CDK1 | Cyclin-dependent kinase 1 | NM_001786 |
| CDC20 | Cell division cycle 20 homolog (S. cerevisiae) | NM_001255 |
| CDC34 | Cell division cycle 34 homolog (S. cerevisiae) | NM_004359 |
| CDK2 | Cyclin-dependent kinase 2 | NM_001798 |
| CDK4 | Cyclin-dependent kinase 4 | NM_000075 |
| CDK5R1 | Cyclin-dependent kinase 5, regulatory subunit 1 (p35) | NM_003885 |
| CDK5RAP1 | CDK5 regulatory subunit associated protein 1 | NM_016408 |
| CDK6 | Cyclin-dependent kinase 6 | NM_001259 |
| CDK7 | Cyclin-dependent kinase 7 | NM_001799 |
| CDK8 | Cyclin-dependent kinase 8 | NM_001260 |
| CDKN1A | Cyclin-dependent kinase inhibitor 1A (p21, Cip1) | NM_000389 |
| CDKN1B | Cyclin-dependent kinase inhibitor 1B (p27, Kip1) | NM_004064 |
| CDKN2A | Cyclin-dependent kinase inhibitor 2A (melanoma, p16, inhibits CDK4) | NM_000077 |
| CDKN2B | Cyclin-dependent kinase inhibitor 2B (p15, inhibits CDK4) | NM_004936 |
| CDKN3 | Cyclin-dependent kinase inhibitor 3 | NM_005192 |
| CHEK1 | CHK1 checkpoint homolog (S. pombe) | NM_001274 |
| CHEK2 | CHK2 checkpoint homolog (S. pombe) | NM_007194 |
| CKS1B | CDC28 protein kinase regulatory subunit 1B | NM_001826 |
| CKS2 | CDC28 protein kinase regulatory subunit 2 | NM_001827 |
| CUL1 | Cullin 1 | NM_003592 |
| CUL2 | Cullin 2 | NM_003591 |
| CUL3 | Cullin 3 | NM_003590 |
| DDX11 | DEAD/H (Asp-Glu-Ala-Asp/His) box polypeptide 11 | NM_004399 |
| DNM2 | Dynamin 2 | NM_004945 |
| E2F4 | E2F transcription factor 4, p107/p130-binding | NM_001950 |
| GADD45A | Growth arrest and DNA-damage-inducible, alpha | NM_001924 |
| GTF2H1 | General transcription factor IIH, polypeptide 1, 62kDa | NM_005316 |
| GTSE1 | G-2 and S-phase expressed 1 | NM_016426 |
| HERC5 | Hect domain and RLD 5 | NM_016323 |
| HUS1 | HUS1 checkpoint homolog (S. pombe) | NM_004507 |
| KNTC1 | Kinetochore associated 1 | NM_014708 |
| KPNA2 | Karyopherin alpha 2 (RAG cohort 1, importin alpha 1) | NM_002266 |
| MAD2L1 | MAD2 mitotic arrest deficient-like 1 (yeast) | NM_002358 |
| MAD2L2 | MAD2 mitotic arrest deficient-like 2 (yeast) | NM_006341 |
| MCM2 | Minichromosome maintenance complex component 2 | NM_004526 |
| MCM3 | Minichromosome maintenance complex component 3 | NM_002388 |
| MCM4 | Minichromosome maintenance complex component 4 | NM_005914 |
| MCM5 | Minichromosome maintenance complex component 5 | NM_006739 |
| MKI67 | Antigen identified by monoclonal antibody Ki-67 | NM_002417 |
| MNAT1 | Menage a trois homolog 1, cyclin H assembly factor (Xenopus laevis) | NM_002431 |
| MRE11A | MRE11 meiotic recombination 11 homolog A (S. cerevisiae) | NM_005590 |
| NBN | Nibrin | NM_002485 |
| PCNA | Proliferating cell nuclear antigen | NM_182649 |
| RAD1 | RAD1 homolog (S. pombe) | NM_002853 |
| RAD17 | RAD17 homolog (S. pombe) | NM_002873 |
| RAD51 | RAD51 homolog (S. cerevisiae) | NM_002875 |
| RAD9A | RAD9 homolog A (S. pombe) | NM_004584 |
| RB1 | Retinoblastoma 1 | NM_000321 |
| RBBP8 | Retinoblastoma binding protein 8 | NM_002894 |
| RBL1 | Retinoblastoma-like 1 (p107) | NM_002895 |
| RBL2 | Retinoblastoma-like 2 (p130) | NM_005611 |
| RPA3 | Replication protein A3, 14kDa | NM_002947 |
| SERTAD1 | SERTA domain containing 1 | NM_013376 |
| SKP2 | S-phase kinase-associated protein 2 (p45) | NM_005983 |
| SUMO1 | SMT3 suppressor of mif two 3 homolog 1 (S. cerevisiae) | NM_003352 |
| TFDP1 | Transcription factor Dp-1 | NM_007111 |
| TFDP2 | Transcription factor Dp-2 (E2F dimerization partner 2) | NM_006286 |
| TP53 | Tumor protein p53 | NM_000546 |
| UBA1 | Ubiquitin-like modifier activating enzyme 1 | NM_003334 |
| B2M | Beta-2-microglobulin | NM_004048 |
| HPRT1 | Hypoxanthine phosphoribosyltransferase 1 | NM_000194 |
| RPL13A | Ribosomal protein L13a | NM_012423 |
| GAPDH | Glyceraldehyde-3-phosphate dehydrogenase | NM_002046 |
| ACTB | Actin, beta | NM_001101 |
| HGDC | Human Genomic DNA Contamination | SA_00105 |
| RTC | Reverse Transcription Control | SA_00104 |
| RTC | Reverse Transcription Control | SA_00104 |
| RTC | Reverse Transcription Control | SA_00104 |
| PPC | Positive PCR Control | SA_00103 |
| PPC | Positive PCR Control | SA_00103 |
| PPC | Positive PCR Control | SA_00103 |
