# Supplementary material for: Improvement of cardiac function by placenta-derived mesenchymal stem cells does not require permanent engraftment and is independent of the insulin signaling pathway
Source: Stem Cell Res Ther. 2014 Aug 21;5(4):102. doi: 10.1186/scrt490 (PMC4354978; doi:10.1186/scrt490)
Supplement: Supplementary file 4 — Additional file 4: Genes analyzed by the insulin signaling pathway polymerase chain reaction (PCR) array. (DOCX 119 KB) [file 13287_2014_412_MOESM4_ESM.docx]

Additional File 4. Genes analyzed by the insulin signaling pathway PCR array.

| **Symbol** | **Description** | **Accession number** |
| --- | --- | --- |
| Acaca | Acetyl-Coenzyme A carboxylase alpha | NM_133360 |
| Acox1 | Acyl-Coenzyme A oxidase 1, palmitoyl | NM_015729 |
| Cfd | Complement factor D (adipsin) | NM_013459 |
| Adra1d | Adrenergic receptor, alpha 1d | NM_013460 |
| Aebp1 | AE binding protein 1 | NM_009636 |
| Akt1 | Thymoma viral proto-oncogene 1 | NM_009652 |
| Akt2 | Thymoma viral proto-oncogene 2 | NM_007434 |
| Akt3 | Thymoma viral proto-oncogene 3 | NM_011785 |
| Araf | V-raf murine sarcoma 3611 viral oncogene homolog | NM_009703 |
| Bcl2l1 | Bcl2-like 1 | NM_009743 |
| Braf | Braf transforming gene | NM_139294 |
| Cap1 | CAP, adenylate cyclase-associated protein 1 (yeast) | NM_007598 |
| Cbl | Casitas B-lineage lymphoma | NM_007619 |
| Cebpa | CCAAT/enhancer binding protein (C/EBP), alpha | NM_007678 |
| Cebpb | CCAAT/enhancer binding protein (C/EBP), beta | NM_009883 |
| Dok1 | Docking protein 1 | NM_010070 |
| Dok2 | Docking protein 2 | NM_010071 |
| Dok3 | Docking protein 3 | NM_013739 |
| Dusp14 | Dual specificity phosphatase 14 | NM_019819 |
| Eif2b1 | Eukaryotic translation initiation factor 2B, subunit 1 (alpha) | NM_145371 |
| Eif4ebp1 | Eukaryotic translation initiation factor 4E binding protein 1 | NM_007918 |
| Ercc1 | Excision repair cross-complementing rodent repair deficiency, complementation group 1 | NM_007948 |
| Fbp1 | Fructose bisphosphatase 1 | NM_019395 |
| Fos | FBJ osteosarcoma oncogene | NM_010234 |
| Mtor | Mechanistic target of rapamycin (serine/threonine kinase) | NM_020009 |
| Frs2 | Fibroblast growth factor receptor substrate 2 | NM_177798 |
| Frs3 | Fibroblast growth factor receptor substrate 3 | NM_144939 |
| G6pc | Glucose-6-phosphatase, catalytic | NM_008061 |
| G6pc2 | Glucose-6-phosphatase, catalytic, 2 | NM_021331 |
| Gab1 | Growth factor receptor bound protein 2-associated protein 1 | NM_021356 |
| Gck | Glucokinase | NM_010292 |
| Gpd1 | Glycerol-3-phosphate dehydrogenase 1 (soluble) | NM_010271 |
| Grb2 | Growth factor receptor bound protein 2 | NM_008163 |
| Grb10 | Growth factor receptor bound protein 10 | NM_010345 |
| Gsk3b | Glycogen synthase kinase 3 beta | NM_019827 |
| Hk2 | Hexokinase 2 | NM_013820 |
| Hras1 | Harvey rat sarcoma virus oncogene 1 | NM_008284 |
| Igf1r | Insulin-like growth factor I receptor | NM_010513 |
| Igf2 | Insulin-like growth factor 2 | NM_010514 |
| Igfbp1 | Insulin-like growth factor binding protein 1 | NM_008341 |
| Ins1 | Insulin I | NM_008386 |
| Insl3 | Insulin-like 3 | NM_013564 |
| Irs1 | Insulin receptor substrate 1 | NM_010570 |
| Irs2 | Insulin receptor substrate 2 | NM_001081212 |
| Jun | Jun oncogene | NM_010591 |
| Kras | V-Ki-ras2 Kirsten rat sarcoma viral oncogene homolog | NM_021284 |
| Ldlr | Low density lipoprotein receptor | NM_010700 |
| Lep | Leptin | NM_008493 |
| Map2k1 | Mitogen-activated protein kinase kinase 1 | NM_008927 |
| Mapk1 | Mitogen-activated protein kinase 1 | NM_011949 |
| Nck1 | Non-catalytic region of tyrosine kinase adaptor protein 1 | NM_010878 |
| Nos2 | Nitric oxide synthase 2, inducible | NM_010927 |
| Npy | Neuropeptide Y | NM_023456 |
| Pck2 | Phosphoenolpyruvate carboxykinase 2 (mitochondrial) | NM_028994 |
| Pdpk1 | 3-phosphoinositide dependent protein kinase 1 | NM_011062 |
| Pik3ca | Phosphatidylinositol 3-kinase, catalytic, alpha polypeptide | NM_008839 |
| Pik3cb | Phosphatidylinositol 3-kinase, catalytic, beta polypeptide | NM_029094 |
| Pik3r1 | Phosphatidylinositol 3-kinase, regulatory subunit, polypeptide 1 (p85 alpha) | NM_001024955 |
| Pik3r2 | Phosphatidylinositol 3-kinase, regulatory subunit, polypeptide 2 (p85 beta) | NM_008841 |
| Pklr | Pyruvate kinase liver and red blood cell | NM_013631 |
| Pparg | Peroxisome proliferator activated receptor gamma | NM_011146 |
| Ppp1ca | Protein phosphatase 1, catalytic subunit, alpha isoform | NM_031868 |
| Prkcc | Protein kinase C, gamma | NM_011102 |
| Prkci | Protein kinase C, iota | NM_008857 |
| Prkcz | Protein kinase C, zeta | NM_008860 |
| Prl | Prolactin | NM_011164 |
| Ptpn1 | Protein tyrosine phosphatase, non-receptor type 1 | NM_011201 |
| Ptprf | Protein tyrosine phosphatase, receptor type, F | NM_011213 |
| Raf1 | V-raf-leukemia viral oncogene 1 | NM_029780 |
| Retn | Resistin | NM_022984 |
| Rps6ka1 | Ribosomal protein S6 kinase polypeptide 1 | NM_009097 |
| Rras | Harvey rat sarcoma oncogene, subgroup R | NM_009101 |
| Rras2 | Related RAS viral (r-ras) oncogene homolog 2 | NM_025846 |
| Serpine1 | Serine (or cysteine) peptidase inhibitor, clade E, member 1 | NM_008871 |
| Shc1 | Src homology 2 domain-containing transforming protein C1 | NM_011368 |
| Slc27a4 | Solute carrier family 27 (fatty acid transporter), member 4 | NM_011989 |
| Slc2a1 | Solute carrier family 2 (facilitated glucose transporter), member 1 | NM_011400 |
| Sorbs1 | Sorbin and SH3 domain containing 1 | NM_009166 |
| Sos1 | Son of sevenless homolog 1 (Drosophila) | NM_009231 |
| Srebf1 | Sterol regulatory element binding transcription factor 1 | NM_011480 |
| Tg | Thyroglobulin | NM_009375 |
| Klf10 | Kruppel-like factor 10 | NM_013692 |
| Ucp1 | Uncoupling protein 1 (mitochondrial, proton carrier) | NM_009463 |
| Vegfa | Vascular endothelial growth factor A | NM_009505 |
