# Supplementary figures and images for: Improvement of cardiac function by placenta-derived mesenchymal stem cells does not require permanent engraftment and is independent of the insulin signaling pathway
Source: Stem Cell Res Ther. 2014 Aug 21;5(4):102. doi: 10.1186/scrt490 (PMC4354978; doi:10.1186/scrt490)

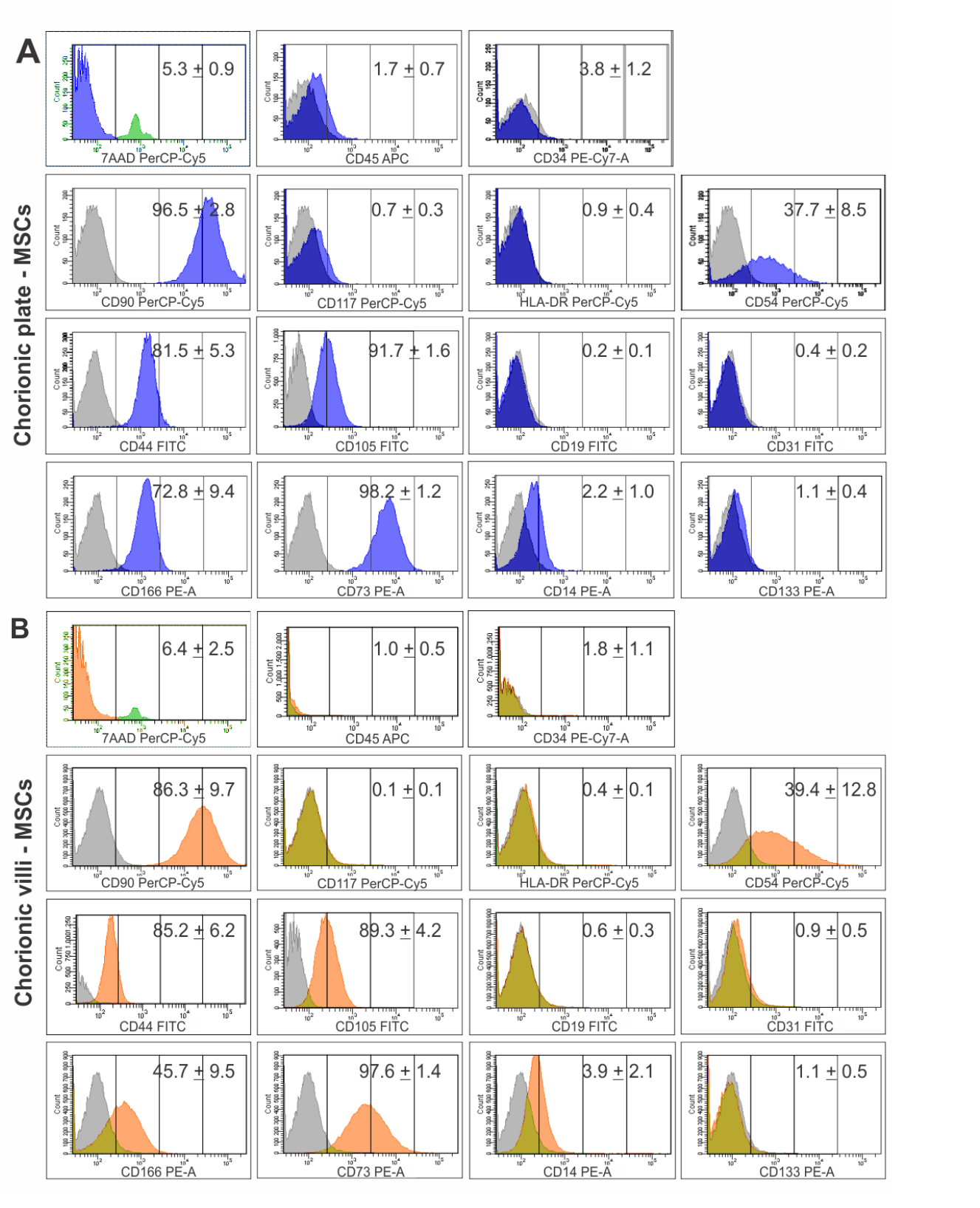

Supplement: Supplementary file 6 — Additional file 6: Flow cytometry analysis. Representative flow cytometry histograms of surface molecule expression in chorionic plate mesenchymal stem cells (cp-MSCs) (A) and chorionic villi mesenchymal stem cells (cv-MSCs) (B). The fluorescence intensity for each molecule is shown in the x-axis. Isotype controls are represented by the light gray curve. Positive events were calculated by subtracting the events obtained using the primary antibody from the isotype control. The average percentage of positive events ± standard error of the mean (SEM) is shown in the upper right corner of each histogram; 7 aminoactinomyocin D (7AAD) (green) was used to exclude dead cells. (TIFF 1 MB) [file 13287_2014_412_MOESM6_ESM.tiff]

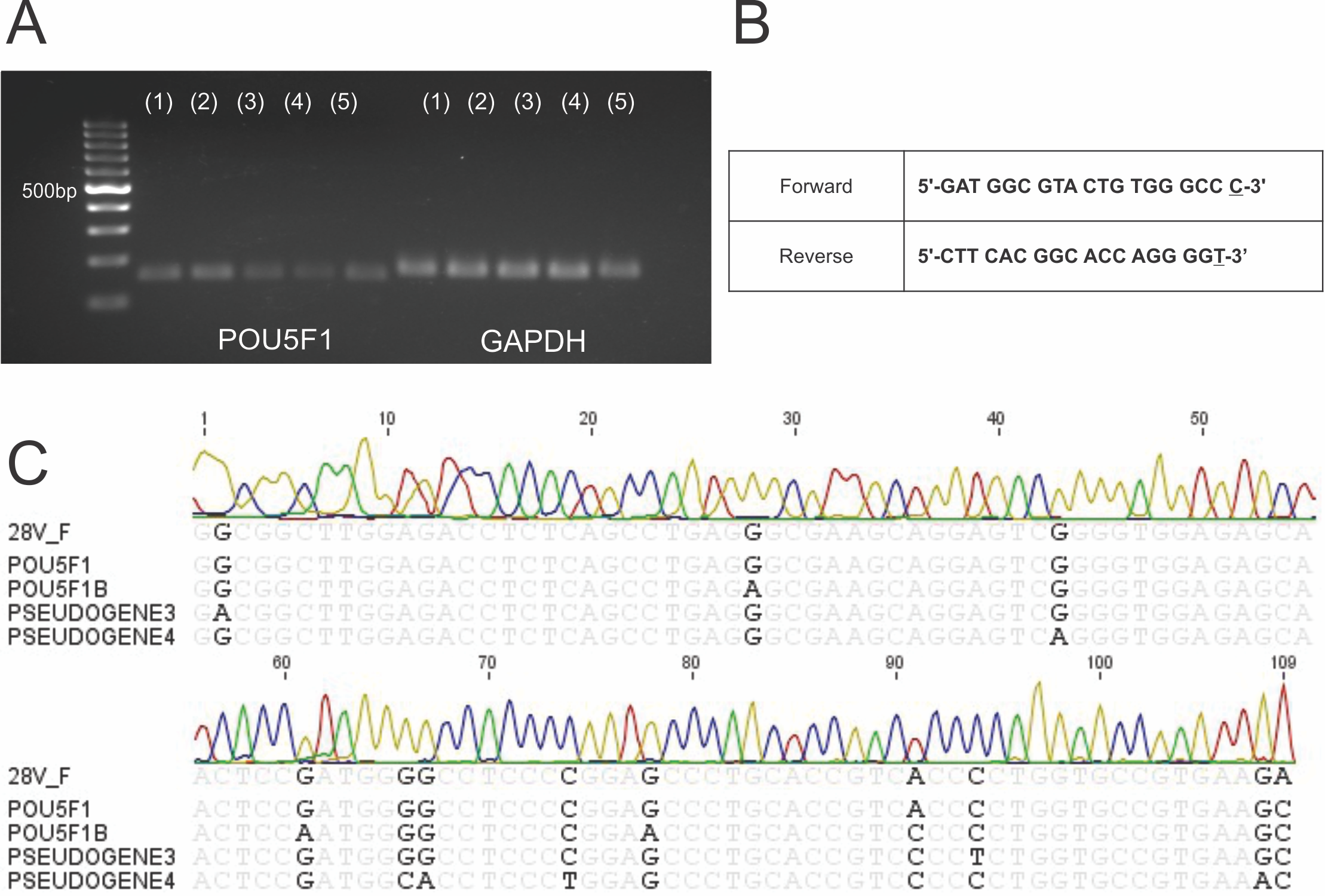

Supplement: Supplementary file 8 — Additional file 8: Expression of POU5F1 (NM_002701.4) in placenta-derived cells. (A) Reverse transcription-polymerase chain reaction (RT-PCR) detection of transcripts POU5F1 (136 bp) and GAPDH (162 bp) in chorionic plate mesenchymal stem cells (cp-MSCs) (lanes 1 and 3), chorionic villi mesenchymal stem cells (cv-MSCs) (lanes 2 and 4), and human embryonic stem cells (lane 5). Samples in lanes 1 and 2 were derived from chorionic plate and chorionic villi obtained from the same placenta. The same is true for samples in lanes 3 and 4. (B) Since the expression of POU5F1 in adult MSCs is controversial [67, 68], we designed primers that recognize transcript variant 1 of POU5F1 but that do not recognize transcript variants 2 and 3, which are not expressed in pluripotent stem cells. Moreover, to differentiate POU5F1 from POU5F1B (NM_001159542.1), which is a different gene not related to pluripotency, both primers have a mismatch in the last nucleotide (underlined), which prevents amplification of POU5F1B. (C) To further confirm our results, PCR products were sequenced and compared with POU5F1 transcript variant 1, POU5F1B, and pseudogenes 3 and 4. Light gray bases show similarities between sequences. Black bases represent mismatches. The PCR product sequence (28V_F) shows 100% similarity only to POU5F1 transcript variant 1. Thus, sequence alignment analysis revealed that adult MSCs express transcript variant 1 of POU5F1. Nevertheless, it is likely that other transcript variants (2 and 3), POU5F1B and/or pseudogenes are also expressed. We immunostained placenta-derived MSCs and detected the presence of nuclear OCT4 protein (data not shown). However, OCT4 (product of POU5F1) has 96% homology to OCT4B (product of POU5F1B) [68], making it impossible to discriminate between them with commercially available antibodies. Finally, it is difficult to speculate which function POU5F1 might have in these cells since they are not pluripotent. (TIFF 2 MB) [file 13287_2014_412_MOESM8_ESM.tiff]

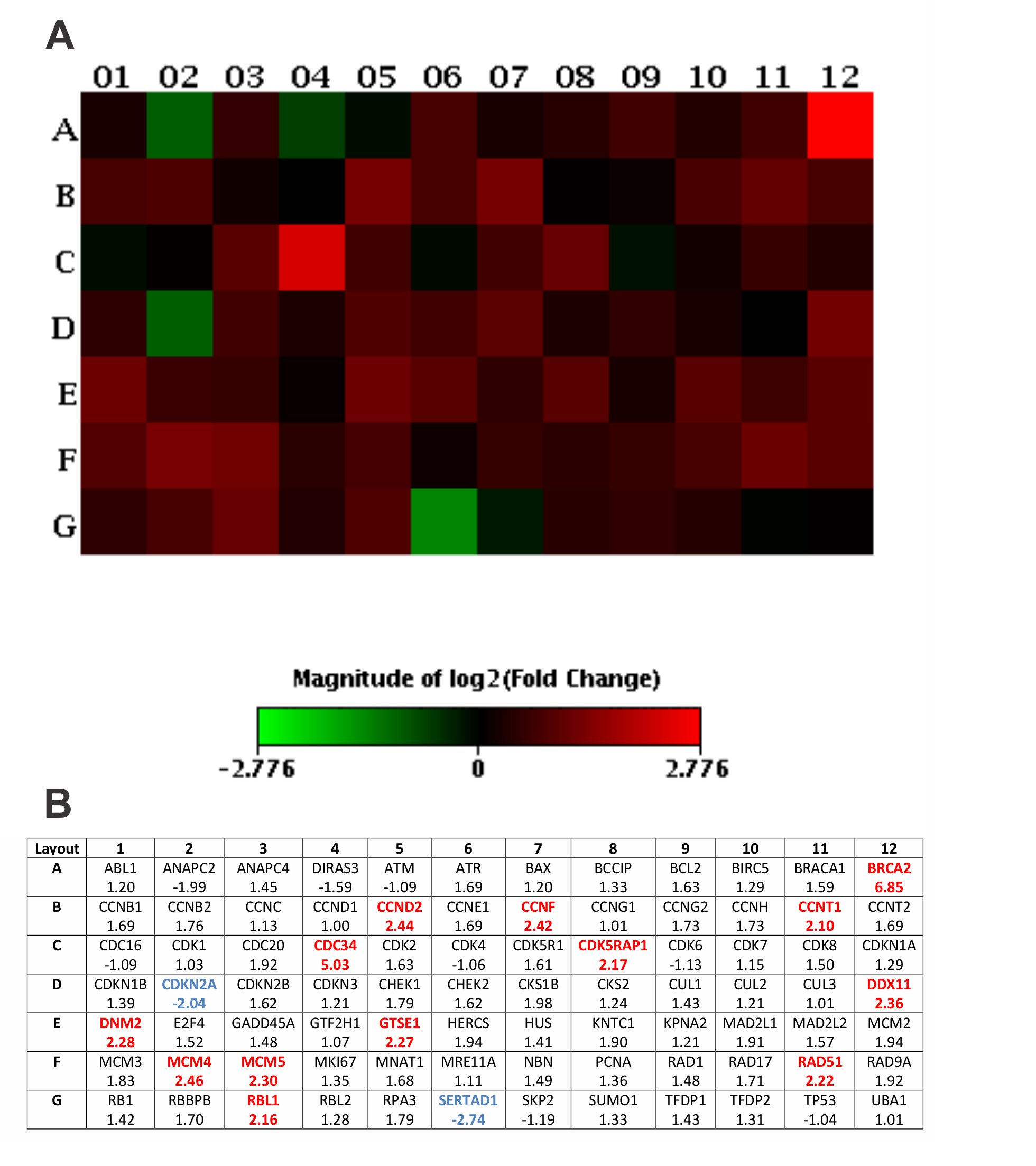

Supplement: Supplementary file 9 — Additional file 9: Expression of cell cycle-related genes in chorionic plate mesenchymal stem cells (cp-MSCs) compared with chorionic villi mesenchymal stem cells (cv-MSCs). (A) Heat map shows log2 (fold-change) of downregulated (green) and upregulated (red) genes in cp-MSCs when compared with cv-MSCs. (B) Table identifies and specifies mean fold-change values for each of the genes analyzed by quantitative reverse transcription-polymerase chain reaction (qRT-PCR). Targets with at least twofold upregulation are shown in red, whereas targets with at least twofold downregulation are shown in blue. Full names and accession numbers for all the genes can be found in Additional file 3. (TIFF 822 KB) [file 13287_2014_412_MOESM9_ESM.tiff]

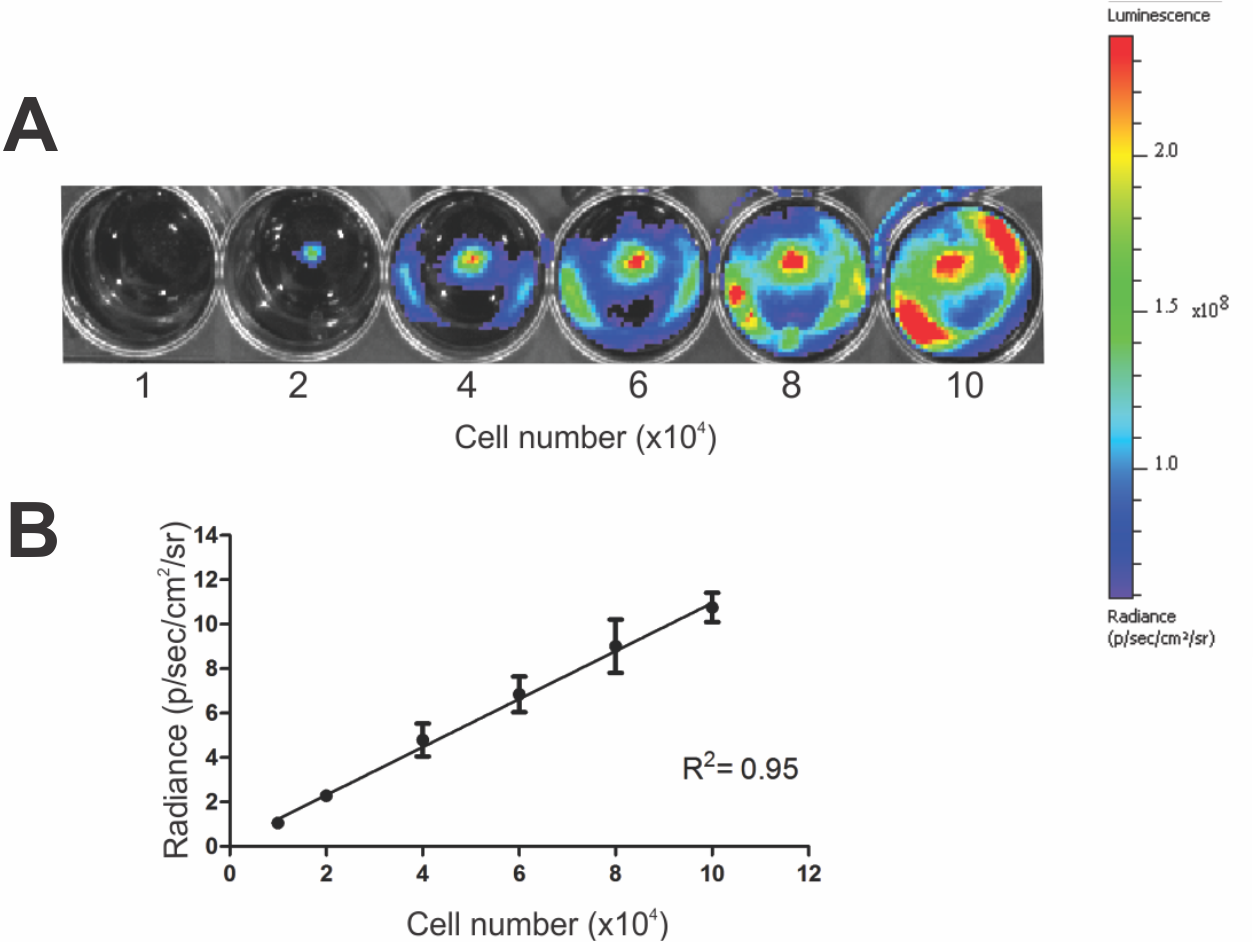

Supplement: Supplementary file 10 — Additional file 10: Luciferase 2 expression in transduced chorionic plate mesenchymal stem cells (cp-MSCs). (A) Bioluminescence imaging of cp-MSCs in vitro shows higher luminescent signal with the increase of cell density. On the right, a scale of the luminescent signal is shown using radiance units. (B) Graph shows a linear correlation between cell numbers and the emitted radiance (R2 = 0.95). (TIFF 542 KB) [file 13287_2014_412_MOESM10_ESM.tiff]

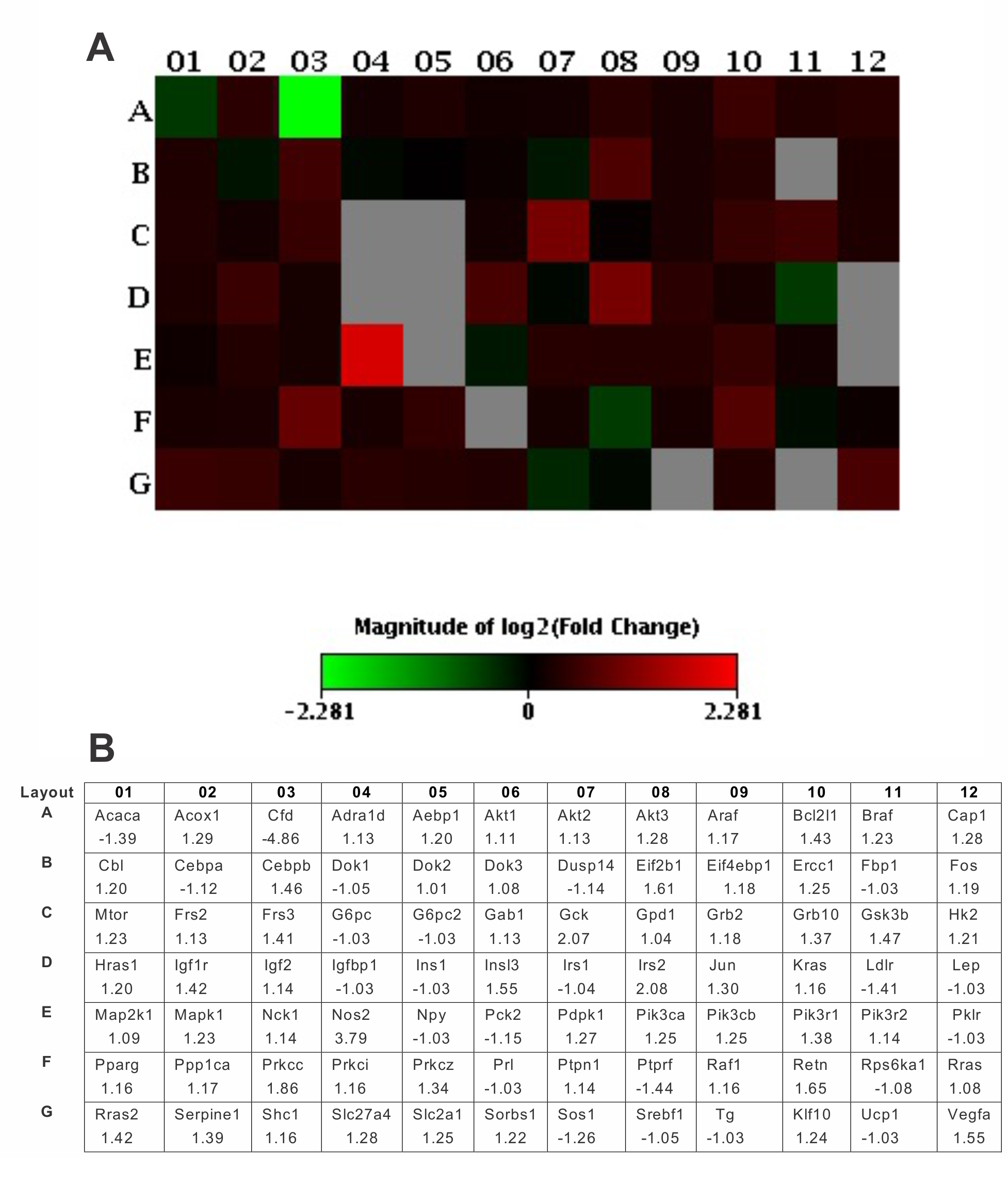

Supplement: Supplementary file 11 — Additional file 11: Expression of insulin signaling pathway-related genes in the hearts of chorionic plate mesenchymal stem cell (cpMSC)-treated compared with placebo-treated mice. (A) Heat map shows log2 (fold-change) of downregulated (green) and upregulated (red) genes in cp-MSCs when compared with placebo-treated mice. (B) Table identifies and specifies mean fold-change values for each of the genes analyzed by quantitative reverse transcription-polymerase chain reaction (qRT-PCR). The vast majority of the genes had less than twofold difference between experimental groups. The four cases with more than twofold difference (Cfd, Gck, Irs2, and Nos2) did not reach statistical significance. Full names and accession numbers for all the genes can be found in Additional file 4. (TIFF 1 MB) [file 13287_2014_412_MOESM11_ESM.tiff]
